# Role of Non-Residential Larval Habitats in *Aedes* Spatiotemporal Egg Production

**DOI:** 10.3390/life14081013

**Published:** 2024-08-15

**Authors:** Julio D. Soto-López, Manuel A. Barrios-Izás, María Carmen Vieira Lista, Antonio Muro

**Affiliations:** 1 Infectious and Tropical Diseases Research Group (e-INTRO), Biomedical Research Institute of Salamanca-Research Centre for Tropical Diseases (IBSAL-CIETUS), Faculty of Pharmacy, University of Salamanca, 37008 Salamanca, Spain; jdjuliosoto@usal.es (J.D.S.-L.); carmelilla@usal.es (M.C.V.L.); 2 Research Institute, University Center of Zacapa, University of San Carlos of Guatemala, Zacapa 01019, Guatemala; manuelbarriosgt@gmail.com

**Keywords:** *Aedes*, arbovirus, density, dengue, distribution, vector control

## Abstract

*Aedes* mosquitoes play a pivotal role as vectors of several arboviral diseases, presenting significant public health challenges worldwide. Their invasive success in tropical regions has raised substantial medical concerns. In Guatemala, *Aedes* mosquitoes are widely distributed and are the primary vectors of the dengue virus. Efforts to control and monitor *Aedes* populations have evolved over time, incorporating strategies such as spatial repellents, larvicides, genetic modifications, and targeted interventions. Previous research has shown the heterogeneous spatial-temporal distribution of these mosquitoes within each season, influenced by temperature variations and favorable environmental conditions for breeding. This study analyzed hot-spot patterns of spatiotemporal egg density in Santa Elena de la Cruz, Petén, Guatemala, from March to September 2022. The aim was to determine whether these patterns were influenced by non-residential larval habitats with plant cover that are not treated by healthcare entities, as well as the proximity between such habitats. Our findings include the collection and registration of over 16,000 *Aedes* eggs during the study period. Local analyses revealed hot-spot patterns in egg densities associated with non-residential larval habitats and their proximity. These insights highlight critical focal points where targeted interventions could be implemented more effectively, resulting in cost-efficient mosquito vector control.

## 1. Introduction

Despite years of vector control attempts, *Aedes* (Stegomyia) *aegypti* (L.) and *Aedes albopictus* (Skuse) mosquitoes continue to play a pivotal role as primary vectors of arboviral diseases, including dengue, yellow fever, chikungunya, and Zika virus throughout the tropical and subtropical world [1,2,3,4,5]. The global impact of these mosquitoes as invasive species is substantial, attributed to their adaptation to human-related factors such as international travel, trade, socioeconomic conditions, access to water, and ecological niches [6,7]. Over the past 50 years, both species have traversed continents, establishing themselves in diverse regions influenced by human activities, densely populated areas lacking reliable water supplies, proper waste management, adequate sanitation [7], and ideal environmental conditions (i.e., temperature, humidity, and rainfall). Given their invasiveness and potential public health implications, vigilant monitoring and strategic control measures for the distribution of *Aedes* are of paramount importance [8].

The approach to addressing the distribution of *Aedes* vectors of arbovirus in Guatemala has involved in situ collection and ecological niche modeling [9,10,11,12,13,14,15]. Their presence spans nearly the entire territory, encompassing 21 of the country’s 22 departments, except for Totonicapán [9]. The breeding grounds of these mosquitoes are intricately linked to indoor containers used for water storage. During the dry season, these include items like barrels, rubber tires, and metal cans, while the rainy season sees proliferation inside barrels and unused containers [10,11]. Notably, outdoor populations also exist in locations such as schools, churches, factories, city parks, and cemeteries [12,14]. In the Guatemala context, vacant lots emerge as the predominant non-residential larval habitat [9,13]. Vacant lots encompass all land within the Republic’s boundaries that does not fall under common or private ownership nor is legitimately claimed by corporations or legal entities. This category also includes common land abandoned due to the verified dissolution of its communal domain. In this study, the term “non-residential larval habitats” is used to denote such spaces.

Some areas and larval habitats may produce more adult mosquitoes than others, according to field surveys and population genetics studies that provide evidence for the spatial heterogeneity of *Aedes* mosquitoes abundance [16,17]. The control of *Aedes* vectors has undergone extensive discussion, leading to the conclusion that the most effective approach involves a combination of techniques. This includes creating anthropogenic spaces refractory to vectors, enhancing disease control strategies, and optimizing resource management by focal point treatments that are more efficient than random control measures (such as passive surveillance strategies recommended by vector surveillance manuals from the 1990s) [18,19,20,21]. This often depends on the ability to predict areas with high potential presence before they spread to other locations. Therefore, identifying these locations with geographic coordinates holds a logical objective [22,23,24,25].

In Latin American countries, attempts have been made to achieve this by calculating larval and pupal indices by study regions, followed by subsequent risk stratification [26]. These indices exhibit inherent variability in precision, as their reliability as estimators of underlying risk decreases inversely with the population at risk. Urban expansion further complicates the use of indices, mainly due to difficulties in reaching the entire population of at-risk households necessary for index establishment.

An alternative approach to elucidate areas with potential elevated vector presence lies in ecologically relevant models, such as niche models [27]. These models serve to bridge knowledge gaps regarding the potential distribution of the vector by enabling the description of favorable environmental conditions that permit the species to thrive in specific areas without the need for immigration [28,29]. The area classification made by geographical models tends to be homogeneous despite the potential distributions of other organisms.

Heterogeneous distributions are prevalent across individuals, species, and environments [20], frequently observed in many infectious and parasitic diseases where a small number of hosts are most heavily infected while the rest of the population carries no infections [25,30,31]. Unraveling the variables underlying such patterns entails studying the presence of undisturbed spaces, examining individual density, and understanding environmental characteristics that promote the establishment of the studied organisms [24]. In the case of mosquitoes, a common practice to identify regions generating higher quantities of vectors (individual production heterogeneity) is the utilization of statistical models. These models highlight clusters of houses yielding disproportionately dense mosquito populations, commonly referred to as “hotspots” [16,22].

Numerous studies employing such methodologies have unveiled the heterogeneous temporal and spatial distribution of dengue-transmitting mosquitoes, highlighting regions prone to producing significantly larger mosquito numbers compared to surrounding ones [24,25,26]. Furthermore, it has been observed that the spatial-temporal distribution of various arboviruses occurs simultaneously, implying that interventions aimed at one disease can impact the prevalence of others [23]. Statistical models and local indicators of spatial autocorrelation serve as valuable tools for identifying areas with high (hotspot) or low (cold spot) values of a continuous variable, such as the number of eggs per ovitrap [26]. One notable statistical model is the local Getis-Ord analysis [Gi*(d)], which gauges the concentration of spatial event values. This analysis aids in calculating local variations in the mean of a variable, thereby pinpointing areas of interest [16].

Given the significant medical implications posed by these disease vectors, there is a heightened interest in identifying the ecological factors influencing the distribution and density of *Aedes* populations. To establish the presence of locations generating higher *Aedes* populations (individual production heterogeneity) over time and space, we analyzed vector egg density patterns from March to September 2022 in Santa Elena de la Cruz, Petén, Guatemala. Furthermore, we assessed whether the existence of these patterns was influenced by nearby non-residential larval habitats.

## 2. Materials and Methods

**Figure 1:**
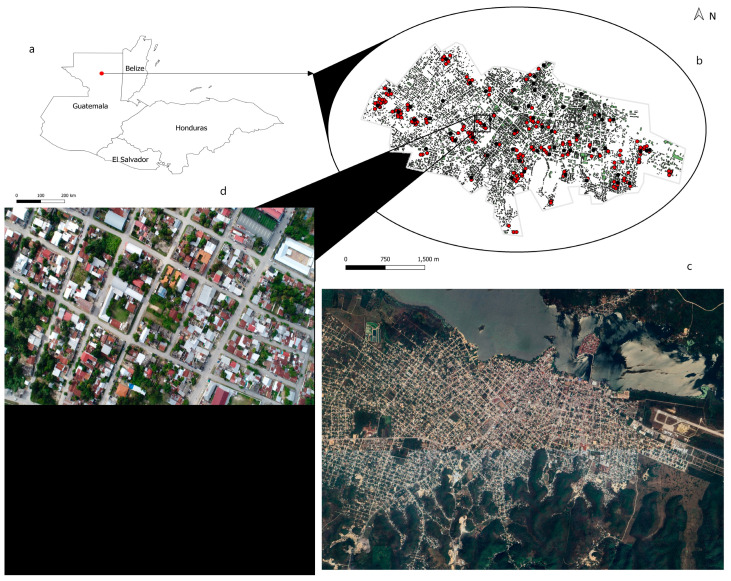
Study area (**a**) administrative boundaries of Guatemala, Belize, Honduras, and El Salvador (**b**) Santa Elena de la Cruz, Petén (**c**) satellite image of panel (**b**), (**d**) closeup of collection area. The black dots show the egg collection coordinates through the installation of ovitraps. The red dots show the localities that were established as non-residential larval habitats with plant cover.

The study area encompassed the municipality of Santa Elena de la Cruz, Petén, situated at the geographical coordinates 16°55′02″ N 89°53′56″ W (Figure 1). This area, along with the city of Flores, serves as the departmental capital of Petén. Santa Elena de la Cruz spans approximately 4336 km^2^, housing around 90,000 inhabitants, and experiences a tropical climate. The average annual temperature recorded in Santa Helena de la Cruz is 25.5 °C, and the approximate precipitation is 1334 mm. Precipitation varies by 225 mm between the driest month and the wettest month. The rainy season starts between the months of June and July and ends at the beginning of the dry season between the months of December and January. The highest relative humidity is measured in October (84.93%), the lowest in April (56.07%). The region is predominantly rural, with over 79% of the population residing in rural areas, characterized by limited access to basic services and a high poverty rate, even below the national average (3%). As part of the research project DIGI 4.8.58.0.74 code B3CU-2022, authorization was obtained from the Petén Norte Health Area Directorate to conduct sample collection activities and to request non-intervention of vector control activities in the non-residential larval habitats selected during the period of our project.

### 2.1. Eggs Collection Process

A total of 60 sampling points were randomly selected using the QuantumGIS random point tool [32] for collecting *Aedes* eggs. Each ovitrap was strategically positioned at least 100 m apart (exceeding the average flight distance of *Aedes aegypti* females [33]) to address sample dependency effects. The eggs of *Aedes* were captured using ovitraps, which were 1-L cylindrical black plastic containers covered with towel-type paper strips measuring 8 cm in width and 35 cm in length. Each container was filled with 0.5 L of water. The exposed paper extended 6–7 cm above the water level inside the ovitraps, ensuring consistency across all ovitraps. Each ovitrap had holes positioned approximately 12 cm above the ground, accommodating more than 600 milliliters. The ovitraps were installed around the home in sheltered areas, protected from wind, direct sunlight, and rain, between 8:00 a.m. and 10:00 a.m. h. They were always placed no higher than 50 cm above the ground. Even though, for some weeks, the sampling points were inaccessible due to logistic issues (see Supplementary Materials for exact dates), ovitraps were deployed once a week from March to September of 2022 within Santa Elena de la Cruz.

During each visit, the procedure involved replacing the paper lining the interior of the ovitraps with a fresh one. These paper strips were carefully collected and placed in individual plastic bags for transportation to the laboratory. Eggs adhering to the paper strips were quantified using a stereo microscope Leica EZ4E (Leica Microsystems, Wetzlar, Germany). After taxonomic assessment using both a stereo microscope and picture keys [34,35], all collected eggs were stored in semi-sterile 1.5 mL vials to isolate them from RNases. Storage was maintained at −20 °C within the facilities of the Centro Universitario de Zacapa (CUNZAC) molecular biology laboratory. Recorded results from positive ovitrap occurrences and corresponding egg quantities were systematically stored in an electronic data sheet. Geographic information was projected onto the GTM Coordinate Reference System, using the QuantumGIS Geographic Information System (Qgis3) for the data projection process [32].

### 2.2. Larvae Collection Process

Thirty-five non-residential larval habitats adjacent to the sampling points were randomly selected for this study due to limitations on funding and personnel. These parcels were characterized by grass, shrubbery, and tree cover. Each of these spaces underwent examination for the presence of the vector, employing a man-hour search approach. The search sequence followed a clockwise direction. All containers suspected to contain *Aedes* larvae found by one person in one hour were scrutinized, and any deposits bearing evidence of the vector’s presence were duly marked. Upon identification, we took the three-stage and fourth-stage larvae from each container using 10-milliliter plastic pipettes. The collected larvae were then carefully transferred into one-milliliter flasks containing 70% alcohol. Each flask was labeled, encompassing geographic coordinates, the source of the sample, the collector’s name, and the collection date.

Subsequently, the collected material was transported to the laboratory for further analysis. Genera identification of the specimens was undertaken using established taxonomic keys [36,37], alongside references provided by the vector-borne disease program of the Ministry of Health. The larval collections were conducted from the first week of October to the last week of November 2022.

Geographic coordinates were documented, complemented by the capture of photographs of the sites (Figure 1). The photographs were taken once between the months of October and November. Additionally, aerial reconnaissance was made with a Phantom 4 drone, maintaining an elevation of 450 m above sea level and allowing us to obtain images in jpg format capturing the study area. The photographs had a 60% to 70% overlap between locations visited. The geospatial coordinates of the drone images were then transferred to QuantumGIS3 (QGIS3) using Python and Folium in a Jupyter Notebook to establish the swept area and extract the photographs corresponding to the coordinates of non-residential larval habitats. Afterward, we calculated the Green-Red Vegetation Index and the Visible Atmospherically Resistant Index of the pictures in QGIS3 to select the non-residential larval habitats with plant cover, as previously described [38]. We also obtained precipitation data from the Instituto Nacional de Sismología, Vulcanología, Meteorología e Hidrología from the months of our study.

### 2.3. Cluster Analysis of Inhabited Building

We conducted a cluster analysis to mitigate any potential impact from house grouping. We use the geographic coordinates of each building in Santa Elena Petén provided by Open Street Map services. We compared the observed clustering of houses with the clustering of ovitraps positive for *Aedes* (ecologically viable). The Ripley and Getis K function, implemented through the Kest, lohboot, and envelope functions within the Spatstat package [39], was employed for this purpose. Additionally, the K functions were corrected to account for edge effects [40]. The spatial clustering patterns were thoroughly examined using the R programming environment [41]. Statistical parameters of K(r) (999 permutations) were derived from entomological data and compared against statistical parameters from dwelling information (with distances quantified in meters). House clusters were inferred when the observed K(r) values surpassed the theoretical random distribution of houses, K(r), for a specific distance (r).

Likewise, the same criteria were applied to ovitraps, evaluating whether the observed K(r) value exceeded the distribution of houses at the same distance (r) [16] and the flight range of the mosquito’s adult stage, which can be up to 100 m over their lifespan [40]. In certain scenarios, this range narrows to approximately 30 m, depending on food and breeding site availability [16,42].

### 2.4. Hot-Spots Analysis

To identify recurrent population clusters responsible for elevated egg density (local analysis), we used the number of eggs per ovitrap. This information was interpolated across the surveyed localities on a weekly basis through the implementation of Inverse Distance Weighting (IDW) in QGIS3 [32]. We used a matrix of 1768 squares of 100 mts^2^. Based on these results, local analyses were conducted using the Gi* statistic in the GeoDa tool [43]. To ensure meaningful week-to-week egg number comparison and to neutralize the influence of varying evaluated zone sizes on the number of positive containers, the values were standardized using Z scores: Z = (Ni − μ)/S, where Ni is the number of eggs per area, μ is the mean number of eggs per study area, and S denotes the standard deviation of eggs within the study area. For each area, Z values were transformed to values between 0 and 1 by dividing them into the maximum value obtained in the Z standardization. The Gi* statistic was applied to these values. The resulting distribution was compared against a random distribution with a statistical significance value (*p* < 0.01) by 999 Monte Carlo permutations. This allowed each polygon to be classified as a member of a group or not. Gi*(d) = 0 indicated a random distribution of mosquitoes. In this study, values greater than 2.575 (with a confidence level of 0.01) with 999 Monte Carlo permutations were considered representative polygons, as previously described [16]. The graphical representation of these outcomes was executed and visualized using the design tool in the QGIS3.

### 2.5. Relationship between Hotspot and Non-Residential Larval Habitats

Finally, we propose a relationship between the hot-spot eggs, the non-residential larval habitats with plant cover, and the distances between each parcel, which remained untouched by healthcare personnel during the study, Equation (1). We suggest that the adjacent non-residential areas produce larvae that contribute to the maintenance of hot spots over time, characterized by productive ovitraps. To assess this relationship, a generalized linear multilevel model with a Poisson distribution was used. Additionally, Gaussian process regression was employed to estimate a function governing the covariance between pairs of plots situated at varying distances. This covariance function, in turn, informed the model predicting the number of eggs across plots:

(1) $$Hi=Poisson(\lambda i)\;Hi=\exp\left(k_{terreno\left[i\right]}\right)\alpha \;\frac{L_{i}^{\beta }}{\gamma }\;k\;\sim MVNormal(\left(0,\ldots ,0\right),K)\;K_{ij}=\eta^{2}\;exp\;\left(-\rho^{2}D_{ij}^{2}\right)+\delta_{ij}\sigma^{2}\;\alpha ∼Exponential(1)\;\;\beta ∼Exponential(1)\;\gamma ∼Exponential(1)\;\eta^{2}∼Exponential(1)\;\rho^{2}\;∼Exponential(0.5)$$

where *H* number of eggs observed, in the *I* observation, *λi* number of eggs expected, *L* is the number of larvae, *α* is the rate of increase of larvae, *γ* is the rate of decrease of larvae, *β* is the flexibility of increase of larvae (decreasing returns), *kterreno*[*i*] variation factor that works as a weight in the form of a proportion (*K* = 0 *exp* (0) = 1, expected value of $\alpha \;\frac{L_{i}^{\beta }}{\gamma }$). The a priori probability distribution was regularized except for the variation factor, for which a Gaussian process was used. *K* is the variation factor, whose probability distribution is Gaussian (multilevel) and is conditioned by the multiplication of a matrix of means (all zero) by *K,* a covariance matrix using pairwise distances between non-residential larval habitats with plant cover. *H* is the covariance matrix, *ρ* is the rate of decrease of *K* with distance, *D* is the distance squared (which causes the covariance to decrease faster at intermediate distances), and $\delta_{ij}\sigma^{2}$ is the variance. *ρ*^2^ and *η*^2^ must be positive, so we used exponentials.

### 2.6. Priors and Fixed Effects

Because of the effect of priors on the models and the lack of information about the dynamics of hot-spot maintenance related to non-residential larval habitats, for this analysis, we adopt “uninformative” exponential priors for parameters *α*, *γ*, *β*, *eta*, and *rho*, aiming to produce estimates like those obtained through maximum likelihood inference. In our model, the varying effects are represented by the vector *K*, which has a length of 35 (variable effects), one for each land value. These effects are modeled using an uncentered Gaussian process, with L_SIGMA as the Cholesky decomposition matrix of the SIGMA covariance matrix. We have three traditional fixed effect parameters (*α*, *γ*, *β*) and two fixed effects (*eta* and *rho*) that do not directly affect the response *λ* independently of the *K* units.

### 2.7. Overfitting Avoiding and Software

To avoid overfitting, we fit seven different models to our data. Differences between models were changing the scales of the variables (log10 of eggs), the number of iterations (2000, 3000, 15,000), the original scale of distance matrix (km to meters), the uninformative priors (exp (0.1)), and the control parameters (adapt_delta, stepsize, max_treedepth).

Our model is easily estimated using Markov Chain Monte Carlo (MCMC) techniques. Four Hamiltonian MCMCs were used for the calculation of the posterior probability, 5000 iterations within each chain, and 1000 iterations of samples for warmup. We had to change the control parameters to avoid divergence transitions (adapt_delta: 0.999, stepsize: 0.01, max_treedepth: 12). We fit the models and sample from the posterior distribution of the parameters with the rethinking package in R [44].

## 3. Results

### 3.1. Eggs Collection

**Figure 2:**
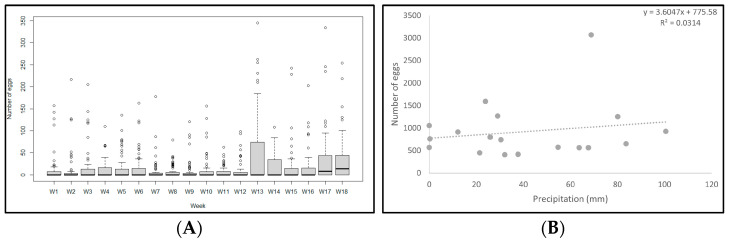
(**A**) Eggs were collected with ovitraps in Santa Elena de la Cruz, Petén, for 18 weeks between March and September. (**B**) Correlation between precipitation and total number of eggs collected per week.

A total of 16,599 eggs were collected over the eighteen weeks. Notably, every container tested showed positive results in at least one of the visits. There was a 300 percent increase in the number of collected eggs starting from the thirteenth week, coinciding with the onset of the rainy season in July (Figure 2) at fifty percent of the sampling points (50th to 100th percentile).

### 3.2. Larvae Collection

**Figure 3:**
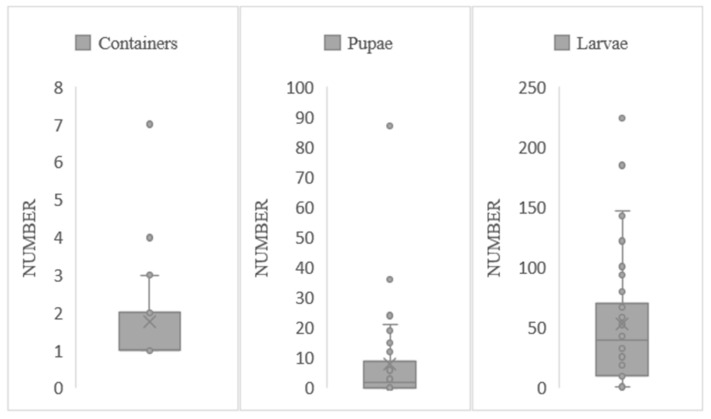
Larvae, containers, and pupae were collected in non-residential larval habitats covered with plants (grass, shrubs, and trees) in Santa Elena de la Cruz Petén in October. The x marks the mean of the number of containers, Pupae, and Larvae. Dots marks the value of the samples.

Non-residential larval habitats were visited, yielding an average value of 1 container per site (Median = 1; Interquartile range = 1), 37 fourth-stage larvae (Median = 37; Interquartile range = 57), and 2 pupae (Median = 2; Interquartile range = 8), per evaluated point (Figure 3).

### 3.3. Inhabited Buildings Clusters Analysis

To address the potential clustering of houses and the interference such patterns might introduce in subsequent analyses, we selected random points within the locality.

Table 1 shows the comparison between the theoretical and observed distance of houses and ovitraps. A value of K greater than the theoretical distance indicates no signs of aggregation in the distribution of houses. Clusters are identified when the observed K values exceed the theoretical K distribution of houses at a given distance. For positive ovitraps, the K(r) values typically surpass the theoretical values associated with houses at distances below one hundred meters. However, the confidence intervals are notably broad within this range, making it challenging to definitively determine the presence of groupings among positive ovitraps. Beyond the one-hundred-meter threshold, the assumption of grouping becomes less evident. This is logical, given that the ovitraps were initially placed at distances exceeding one hundred meters. Consequently, the model’s ability to distinguish groupings below this distance is limited.

At distances exceeding one hundred meters, the distribution of ovitraps shows an absence of distinct groups. The consistently positive results across all sampling points underscore the ecological viability of the ovitraps (Supplementary Data S1). This data pattern reaffirms the effectiveness and reliability of the ovitraps in capturing and assessing female *Aedes* activity.

### 3.4. Eggs Hot-Spot Analysis

**Figure 4:**
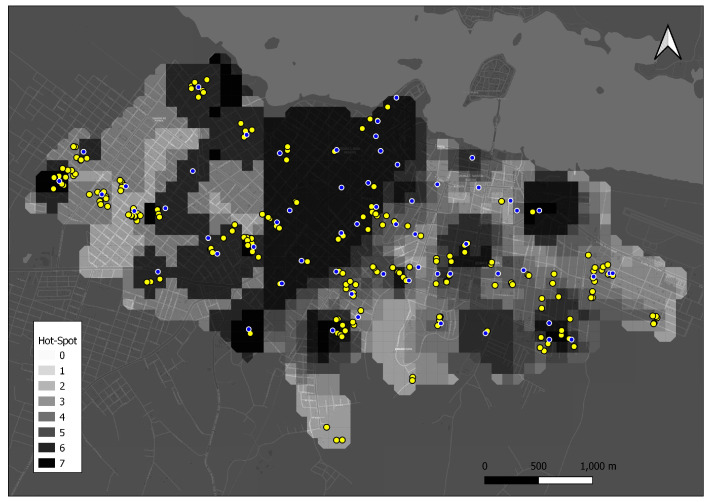
Hot spots were found in the study region. The colors represent the number of weeks in which the colored grid is considered a hot spot (*p* < 0.01). Blue points are the ovitraps positions. Yellow points are non-residential larval habitats with plant cover positions. The resolution of the squares is 100 mts^2^.

Figure 4 shows areas with statistically significant higher egg counts compared to adjacent squares (*p* < 0.01). Specifically, the areas highlighted with six or seven weeks of above-average egg counts are surrounded by non-residential larval habitats that have not been intervened by vector control strategies. The spatial placement of these habitats might impact the persistence of egg densities over time, although the influence of distance remains somewhat ambiguous.

### 3.5. Relation of Non-Residential Larval Habits in Hot-Spot Production: Evaluation of the Model

In Table 2, we compare seven models using the Widely Applicable Information Criterion (WAIC). This criterion provides simple estimates of out-of-sample model accuracy, giving us a rough measure of our model’s flexibility and, therefore, overfitting risk. We observe that the values of efficient approximate leave-one-out (LOO) cross-validation using Pareto smoothed importance sampling (PSIS) correspond with the WAIC, so we used the last criterion to compare our models. We chose the 7th model from different adjusted models after comparing them (lower WAIC and SE).

**Figure 5:**
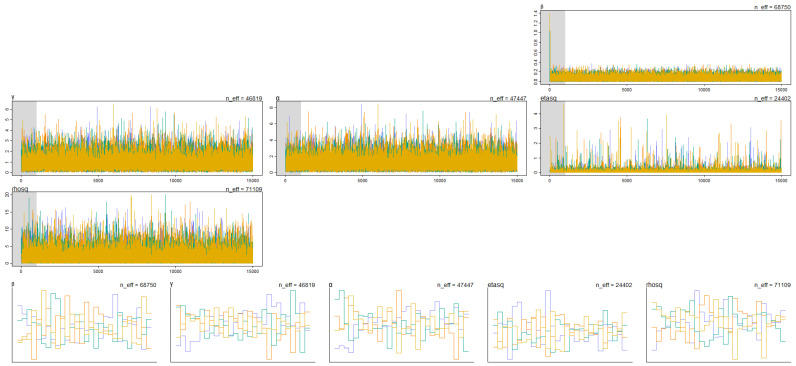
Trace and trank plots of Markov Chain Monte Carlo (MCMC) from four chains defined by the multilevel model.

The trace and trank plots of the four chains from the Markov Chain Monte Carlo (MCMC) traces of the higher model (7) look healthy. Both chains are stationary around the same values (resemble “white noise” around a stable mean), according to their corresponding priors, and mixing is good. There are no wild detours into extreme values, and no single chain consistently stays in one region of the parameter space (Figure 5). We were able to avoid divergent transitions with the changes in the control parameters of the model and with the use of non-informative priors and non-centered parameterization.

### 3.6. Multilevel Model Results

Table 3 shows the plausibility of each parameter value after averaging over the plausibility of each other parameter given by a Gaussian distribution with pointwise mean and standard deviation. The 5.5% and 94.5% correspond to an 89% credible interval (CI). The Rhat ratio of all the parameters calculated by the multilevel model shows that total variance shrinks to average variance within the four chains used. The effective number of samples in all the parameters is far above 20,000, which is plenty for accurate inference of the posterior.

The parameter for the rate of decrease of larvae (*γ*) has a mean value of 0.88. The compatibility interval (0.16, 2.07) shows that plausible values of *g* can vary quite a bit, although, in general, they seem to be concentrated at values less than 2.07. The parameter for the rate of increase of larvae (*α*) has a mean value of 1.12 with a considerable standard deviation of 0.81, indicating high uncertainty. The compatibility interval (0.20, 2.63) shows a wide range of plausible values, suggesting that a could have a significant effect. The parameter for the flexibility of increase of larvae (*β*) has a mean value of 0.07, with a standard deviation of 0.05, indicating low uncertainty, and the compatibility interval (0.01, 0.17) suggests that the effect of larvae on λ is small but positive. The low mean and small compatibility interval of the *eta* parameter suggest that the variability between the *k* effects is not very high. The rho parameter has a mean of 1.74 and a high standard deviation of 1.92, indicating considerable uncertainty. Since we expect eggs to vary greatly between locations, it is important to consider that the high uncertainty in the *rhos* range of compatibility (0.03, 5.40) suggests that there is a lot of variability in how non-residential larval habitat effects are spatially correlated.

**Figure 6:**
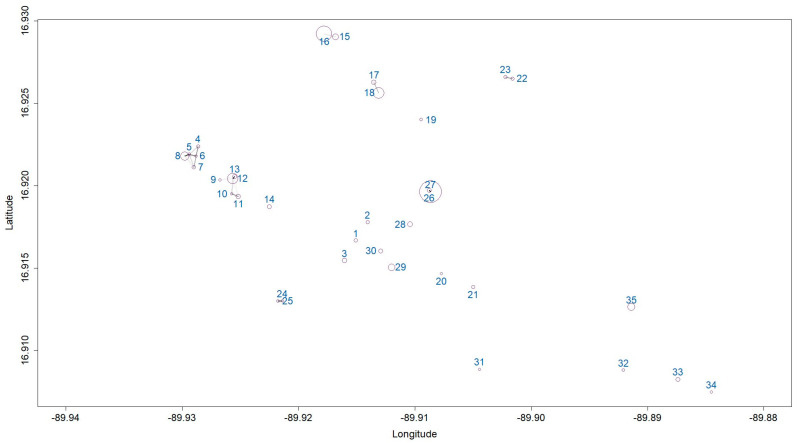
Subsequent correlations between non-residential larval habitats with plant cover in the geographical space studied. The size of the points is represented according to the density of larvae found in the evaluated area. Darker lines indicate more robust correlations, with white lines representing zero correlation and black lines signifying a 100% correlation.

Figure 6 presents a visual representation of the relations found with the multilevel model (Supplementary Materials S2). The numbers represent the non-residential habitats. According to the multilevel approach, the intensity of correlation tends to be stronger among non-residential habitats that are closer to each other. There is a stronger correlation between two groups of non-residential larval habitats, 4–8 and 9–13. The direct impact of these correlations on hot-spot eggs might be challenging to discern, but notable spatial trends are evident.

There is a stronger correlation between non-residential larval habitats within hot-spot areas compared to those in adjacent areas (*ρ*^2^: 0.0802, 0.0037–0.2339 89% CI. *η*^2^: 1.7366, 0.0295–5.3976 89% CI). This observation aligns with the outcomes of our previous hot-spot analyses. These results collectively indicate patterns not only in terms of egg quantities over both time and space but also in the larvae generated within the surrounding vicinity of the hotspots.

**Figure 7:**
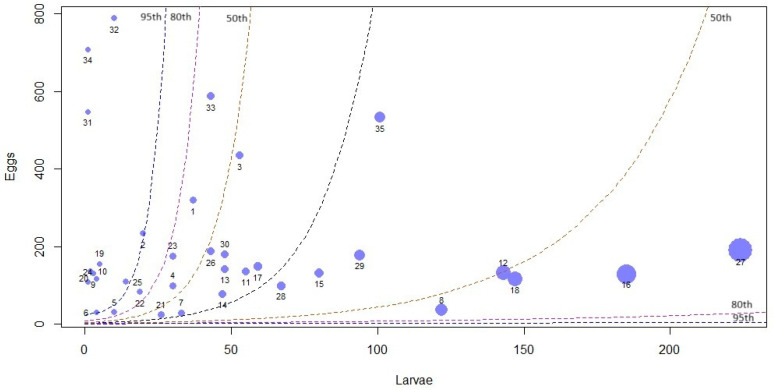
Relation of hot-spot eggs and the number of larvae found in the surrounding non-residential larval habitat. The black line shows the function of the posterior median. Brown, purple, and blue lines show the 50th, 80th, and 95th percentile of the posterior interval. The size of the points is represented according to the density of larvae found in the non-residential larval habitats.

The relationship between non-residential larval habitats and hot spots is depicted in Figure 7. The multilevel model shows how the number of larvae in non-residential habitats is an explanatory framework for understanding hot-spot egg counts (Figure 7) following a Poisson distribution. This suggests a potential link between larval populations in uninhabited areas and the dynamics observed in adjacent regions of higher egg productivity.

According to the fitted model, changes in hot-spot egg counts can follow the dynamics of larvae in non-residential larval habitats (Table 3). Hot-spot areas with strong correlations with non-residential larval habitats (4–8 and 9–13) have egg counts estimated by the model corresponding to the number of larvae found in surrounding parcels (Figure 7) within the 80th percentile of the posterior interval (PI). In Parcel 8, fewer eggs were observed than expected for the number of larvae found. Another strongly spatially correlated group consists of plots 9–13. In this group (4–8), plots 9 and 10 show discrepancies between the observed and expected egg counts based on the number of larvae in the surroundings, according to the model (95th PI). Parcels 31, 32, and 34 (refer to Supplementary Data S1) exhibit cumulative egg counts that surpass the anticipated count based on their respective larval populations (95th PI). We also observed no correlation between these parcels, as shown in Figure 6.

## 4. Discussion

Our study has provided valuable insights into the dynamic and spatially varying nature of arbovirus-transmitting vectors over time and space within a village in Guatemala. Understanding the ecological factors that influence these patterns, as well as the bionomics of these organisms, is pivotal for implementing focal interventions and more effective vector control strategies, indirectly reducing arbovirus transmission. Our results support the existence of a concentrated distribution of *Aedes* eggs and reveal a significant correlation between the presence of non-residential habitat larvae and higher egg counts in adjacent hot-spot areas. This suggests that non-residential larval habitats play a crucial role in sustaining mosquito populations, potentially serving as a primary source of infestation in nearby residential areas.

We identified hot spots that are notably clustered in the central districts of Santa Elena, with sporadic distribution towards the periphery. It is noteworthy that these identified points exhibit stability over time. Hot spots are often situated 150 m away from non-residential larval habitats with favorable conditions for vector persistence, which reinforce the high densities of mosquitoes over time. Non-residential larval habitats can harbor viable eggs due to quiescence mosquitoes (up to one year when desiccated) until suitable conditions recur [45]. The presence of points responsible for elevated vector densities presents an opportunity for targeted intervention strategies in mosquito control and for optimizing resource allocation compared to conventional uniform interventions [26,40]. This approach could effectively reduce the overall mosquito population and the risk of disease transmission.

The correlation between the hot-spots egg density and non-residential larval habitats larvae identified in ecologically viable containers allows us to pinpoint critical locations of high importance in the resilience of *Aedes* egg populations across space. Interestingly, our results also suggest a potential effect of the distance from non-residential larval habitats on the hot-spot number of eggs. Similar effects have been demonstrated in other organisms [20], highlighting the importance of targeted interventions in areas where larval production is significant. While the model generally predicts egg counts based on larval presence in surrounding areas, there are notable discrepancies in certain plots (e.g., Parcel 8, 9, and 10). These discrepancies highlight the complexity of mosquito breeding behaviors and suggest that other environmental or ecological factors might be influencing egg distribution.

Simulation results indicate that interventions targeting hot spots could yield more effective outcomes compared to homogeneous interventions [22]. This aligns with previous proposals advocating for focused efforts in dengue vector control and surveillance, as previously suggested [16,20,22,24], but highlighting a new component in *Aedes* dynamics: non-residential larval habitats. Healthcare facilities can enhance their impact by strategically addressing and intervening in non-residential larval habitats that serve as breeding hotspots for mosquitoes. These strategies should not only focus on residential areas but also consider the broader environmental context, including neglected non-residential zones.

It’s worth noting that we did not measure the relationship between larval numbers and plant composition, which could be an important factor to consider in future research, as previously reported [46,47].

The geographic distance relationship outlined by our results may be influenced by unaccounted variables, such as similarities between geographically proximate terrains. Factors like population density, proximity to commercial areas or shopping centers, distance to waste disposal sites, and the presence of discarded tires could potentially play a role. These variables could contribute to the observed patterns in *Aedes* distribution and density.

Another important consideration is the relatively low survival rate of mosquitoes, with reported rates of around 3% from the egg to adult stage [48,49]. Measuring eggs in the field might not fully capture the risk of dengue transmission posed by adult females. The presence and density of *Aedes* mosquitoes, as gauged through egg counts, do not directly indicate the risk of arbovirus contagion. While this aspect is not the central focus of our study, if a risk indicator of viral transmission is desired, we recommend measuring the population of fed adult female mosquitoes alongside estimates of population age.

Arboviral diseases surpass the capacity of health care institutions to control them, a reality underscored since the reinfestation of *Aedes* aegypti in Guatemala in 1973 [50]. Addressing and mitigating this issue requires multi-sectoral participation. However, the prevailing socioeconomic crisis in most of the at-risk populations constrains the full potential benefits that comprehensive participation from all affected sectors could bring to vector control. Therefore, the utilization of methodologies adapted to the current societal state, influenced by arboviruses, is indispensable for effective vector control and surveillance. Given their demonstrated reliability, ovitraps could be deployed more widely to gather comprehensive data on mosquito distribution and to evaluate the impact of control interventions over larger areas.

While various factors contribute to the distribution of *A. aegypti* and *A. albopictus*, including urbanization and sanitary conditions, climate exerts the most significant influence on species and disease distribution [51]. Although our study does not show a relationship between precipitation and the density of *Aedes* eggs (Figure 2B), it is imperative to emphasize that if current climate trends persist, the expansion of vectors distribution, as well as the viruses they transmit, will likely intensify [52], rendering control efforts more challenging.

It becomes evident that further research is imperative to broaden our understanding of mosquito population dynamics and adaptability. This entails incorporating a wider range of variables and acknowledging their interactions to inform and guide intervention strategies. Healthcare systems of affected countries can prioritize disease control through vector surveillance and management in regions prone to producing significantly larger mosquito numbers compared to surrounding areas. These goals, focusing on spatiotemporal patterns, can be achieved through the utilization of spatial analysis, as we successfully demonstrate. Furthermore, we establish the effect of the proximity of non-residential larval habitats with plant cover on hot-spot patterns as a new variable to consider.

The spatial analysis framework employed in this study holds promise in its potential application to analyze oviposition patterns in other vector mosquitoes, such as *Anopheles* spp. or *Culex* spp. These species may exhibit analogous spatial oviposition patterns contingent on their ecological behaviors. By implementing this approach, healthcare institutions can enhance their effectiveness by strategically targeting and intervening within non-residential larval habitats responsible for mosquito breeding hotspots. This optimization can yield more streamlined and effective control measures and strategies in the ongoing battle against mosquito-borne diseases. The integration of such analyses can catalyze advancements in public health efforts and enhance our ability to mitigate the impact of these diseases on affected populations.

Our findings underscore the importance of considering both residential and non-residential habitats in mosquito control efforts. Seasonal fluctuations in dengue cases, which are well-documented, often mask the underlying patterns of *Aedes* mosquitoes’ activity despite their significance for public health. Presently, the vector control strategy tends to be reactive; however, our findings suggest a shift towards a preventive approach. The significant correlation between larvae in non-residential areas and hot-spot egg numbers underscores the need for comprehensive and targeted vector control strategies aimed at eradicating hotspots and their potential breeding grounds, including uninhabited habitats.

## Figures and Tables

**Table 1:** Results of the reduced second-moment function of Ripley and Getis in Santa Elena, Petén.

R	K(r)	CI_inf_	CI_sup_	K’(r)_H_	CI_inf_	CI_sup_	K(r)_O_	CI_inf_	CI_sup_
25	2032.3	1998.9	2063	3249.1	3215.8	3283.5	6604.9	0	16,512
50	7819.6	7760.5	7878.9	11,668.5	11,578.5	11,759.5	13,209.8	3302.5	26,419
100	31,278.3	31,164.2	31,396.9	42,694.4	42,405	42,949.5	13,209.8	3302.5	26,419
150	71,299	71,079	71,527.5	92,051.2	91,531	92,545.9	40,390.1	19,814	63,507
200	126,342.7	126,059	126,672	156,752.3	155,976	15,752	99,834.3	65,960	135,400

R: Distance in meters, K(r): Theoretical, K’(r)_H_: Observed in houses, K(r)_O_: Observed in positive ovitraps. CI_inf_: Lower confidence interval. CI_sup_: Upper confidence interval. Clusters can be identified through the theoretical distance calculated against the observed distance of the containers and houses studied.

**Table 2:** Gaussian approximations for each parameter’s marginal distribution of the multilevel model and its evaluation by out-of-sample relative K-L divergence.

Model	WAIC	pWAIC	Standar Error
1	127.6	0.7	2.21
2	134.0	1.3	3.46
3	254.1	15.5	28.61
4	182.4	9.2	7.01
5	129.6	1.3	2.21
6	183.1	5.9	25.85
* 7	95.9	1.4	3.79
7	95.9	1.4	3.71

PSIS: efficient approximate leave-one-out (LOO) cross-validation using Pareto smoothed importance sampling (PSIS). WAIC: Widely Applicable Information Criterion. * lppd: log pointwise predictive density. pWAIC: the effective number of parameters.

**Table 3:** Gaussian approximations for each parameter’s marginal distribution of the multilevel model.

	Mean	Standar Deviation	5.50%	94.50%	n_eff	Rhat
*γ*	0.881016996	0.639191811	0.1587952	2.06881852	46,818.543	1.00008543
*β*	0.067488959	0.053101507	0.00541973	0.1676695	68,750.3299	0.99998469
*α*	1.117058114	0.805886235	0.20272539	2.62788381	47,446.6902	1.00013199
*etasq*	0.080203564	0.131349391	0.00374498	0.23398684	24,402.3169	0.99999281
*rhosq*	1.73661865	1.921797854	0.02956985	5.3976698	71,109.0244	0.99999232

n_eff: number of independent samples. Rhat: Gelman-Rubin convergence diagnostic. *γ*: flexibility of increase of larvae. *β*: rate of decrease of larvae. *α*: rate of increase of larvae. *etasq*: covariance matrix. *rhosq*: rate of decrease of *K* with distance.

## Data Availability

All data generated or analyzed during this study are included in this published article (and its Supplementary Information files).

## Supplementary Materials

The following supporting information can be downloaded at: https://www.mdpi.com/article/10.3390/life14081013/s1, Data S1: Data and S2: Parameter estimate information of the model.
